# Gastrointestinal Tract and Dietary Fiber Driven Alterations of Gut Microbiota and Metabolites in Durco × Bamei Crossbred Pigs

**DOI:** 10.3389/fnut.2021.806646

**Published:** 2022-01-28

**Authors:** Guofang Wu, Xianjiang Tang, Chao Fan, Lei Wang, Wenjuan Shen, Shi'en Ren, Liangzhi Zhang, Yanming Zhang

**Affiliations:** ^1^Key Laboratory of Adaptation and Evolution of Plateau Biota, Northwest Institute of Plateau Biology, Chinese Academy of Sciences, Xining, China; ^2^Plateau Livestock Genetic Resources Protection and Innovative Utilization Key Laboratory of Qinghai Province, Qinghai Academy of Animal and Veterinary Medicine, Qinghai University, Xining, China; ^3^Qinghai Provincial Key Laboratory of Animal Ecological Genomics, Northwest Institute of Plateau Biology, Xining, China; ^4^College of Life Sciences, University of Chinese Academy of Sciences, Beijing, China

**Keywords:** dietary fiber, gut bacteria, metabolites, gut segment, Durco × Bamei crossbred pig

## Abstract

Gastrointestinal tract and dietary fiber (DF) are known to influence gut microbiome composition. However, the combined effect of gut segment and long-term intake of a high fiber diet on pig gut microbiota and metabolite profiles is unclear. Here, we applied 16S rRNA gene sequencing and untargeted metabolomics to investigate the effect of broad bean silage on the composition and metabolites of the cecal and jejunal microbiome in Durco × Bamei crossbred pigs. Twenty-four pigs were allotted to four graded levels of DF chow, and the content of jejunum and cecum were collected. Our results demonstrated that cecum possessed higher α-diversity and abundance of Bacteroidetes, unidentified *Ruminococcaceae* compared to jejunum, while jejunum possessed higher abundance of *Lactobacillus, Streptococcus*. DF intake significantly altered diversity of the bacterial community. The abundance of Bacteroidetes and *Turicibacter* increased with the increase of DF in cecum and jejunum respectively. Higher concentrations of amino acids and conjugated bile acids were detected in the jejunum, whereas free bile acids and fatty acids were enriched in the cecum. The concentrations of fatty acids, carbohydrate metabolites, organic acids, 2-oxoadipic acid, and succinate in cecum were higher in the high DF groups. Overall, the results indicate that the composition of bacteria and the microbiota metabolites were distinct in different gut segments. DF had a significant influence on the bacterial composition and structure in the cecum and jejunum, and that the cecal metabolites may further affect host health, growth, and slaughter performance.

## Introduction

The mammalian gastrointestinal tract is a long and connected lumen with distinct structures and functions because of the distinct epithelial cells in different segments (1), which may create distinct microenvironments in the gastrointestinal tract (2). Many studies have reported that the community structure of bacteria colonizing different gut segments displays spatial heterogeneity, which serves distinct functions for the host (3–8). Additionally, the host gut functions in digesting food resources were distinct in different segments of the gastrointestinal tract, resulting in different nutrient supplies for the microbiotas, which may also contribute to the spatial heterogeneity of the bacterial community.

Dietary fiber (DF) influences the gut microbiome composition and plays an important role in improving host health (9–13). With the deepening research on the physical and chemical properties and the physiological role of nutrition, DF increases the potentially probiotics, enhances the constitution, promotes intestinal epithelial barrier function, and maintains homeostasis in the gastrointestinal tract (14, 15). Further, DF is fermented by gut bacteria, producing short chain fatty acids (SCFAs) and other metabolites (16), which in turn, support the growth of probiotics and suppress harmful bacteria in the gut. Subsequently, increasing DF can reduce fat deposition, increase the carcass lean rate, and improve carcass quality (17).

Altered DF intake usually changes the composition of the host gut microbial community, which may induce variation in many types of metabolites (13, 18). Most studies examining the effect of DF on gut microbial metabolites in pigs have mainly focused on SCFAs (10, 12). However, a high-DF diet can indirectly influence the digestibility of energy, amino acids, and other nutrition (19). As most of these small metabolites are closely related to host health and growth performance (10, 18, 20), determining the variation in microbial metabolites across different graded levels of DF diets could add to the available information for improving animal health and production. Liquid chromatography-mass spectrometry (LC-MS) is a popular tool for metabolomics analyses to determine the non-targeted profiles of a vast number of small metabolites. As this technique for non-targeted metabolites is easily available, it has been applied in many fields.

Previous studies have confirmed that bacteria in the gastrointestinal tract of pigs display spatial heterogeneity, and that DF significantly alters the composition and structure of gut bacteria, resulting in variation of metabolites (5, 12). However, relatively few studies have conducted a systematic investigation with respect to the impact of gut segments on the gut microbiome and the metabolites in pigs, the response of gut bacteria to DF in different gut segment microenvironments, and the metabolomics features and related metabolic pathways across different graded levels of DF diets in different gut segments.

Bamei pigs (*Sus scrofa*) are a local breed of livestock in the QingHai Province. After long-term natural and artificial selection these animals have developed crude fiber resistance, strong fat deposition capacity, and good meat quality characteristics (21, 22). Studies have shown that the crude fiber digestibility of Bamei pigs (52.08%) is significantly higher than that of wild pigs (45.72%), indicating that Bamei pigs have a strong ability to withstand rough feed (23). Binary pigs and ternary hybrid pigs have better combining ability and heterosis; thus, hybrid pigs have larger hindquarters and better quality compared to purebred Bamei pigs in breeding performance (24–26). Being at a high altitude, Qinghai province has a cold and dry climate which leads to the feed resources are scarce. It is thus necessary to develop the local feed resources and to use them rationally. Broad bean is one of the important crops on the Qinghai-Tibet Plateau, with advantages such as wide sources and low cost (27). However, the impacts of broad bean on the gut microbiome and metabolites of Durco × Bamei crossbred pigs have been poorly understood.

In this study, we explored the effects of different proportions of a fermented silage broad bean diet on the intestinal microbial flora and metabolites of Bamei binary hybrid pigs, to provide a theoretical basis for implementing the national “grain to feed” policy; thus, we studied the gut microbiome in the cecal and jejunal contents collected from a cohort of individuals on a prescribed diet including control, 10, 17, and 24% of fermented silage broad bean in a Cross-Sectional study design. We then analyzed the compositional variations in the cecal and jejunal microbiota using 16S rRNA gene sequencing. Moreover, to identify the functional potential of the microbiome during DF digestion, we leveraged non-targeted metabolites to determine the complement of metabolites in the cecum and jejunum.

We found that both gut segment and DF were important factors in shaping gut bacterial community composition and structure of Durco × Bamei crossbred pigs. Bacteria colonizing in different gut segments possessed distinct functions in digesting nutrition, resulting in distinct metabolites. Increasing the proportion of DF in diet significantly altered the composition and structure of bacterial community in cecum, resulting in alteration of concentration of their metabolites including bile acids, fatty acids, carbohydrates and carbohydrate conjugate, and organic acids which may potentially play an important role in regulating host nutrition absorbing and health.

## Materials and Methods

### Animals

In total, 24 pigs (Duroc × Bamei pigs) averaging 3 months in age with an initial body weight of about 25.5 kg were obtained from the Qinghai Bamei pig Breeding Farm.

### Study Design

In total, 24 pigs were equally and randomly allotted to four groups (Control, Groups I, II, and III; 6 pigs per group). Three males and three females were assigned to a group. Pigs in the control group were fed basic chow, while the chow of the pigs in groups I, II, and III were supplemented with 10, 17, and 24% silage, respectively. The ingredient composition, nutrient and energy content of the basic diet and the silage were shown in Table 1. The pre-trial period was 7 days and the normal experimental period was 90 days. After the experiment, the pigs were slaughtered to collect their intestinal contents, which were stored at −80°C for later use.

**Table 1:** Ingredient composition and the nutrient and energy content of the diet.

**Item**	**Control**	**Groups**		
		**Group I**	**Group II**	**Group III**
**Material (%)**				
Corn	79.13	70.57	62.38	53.96
Soybean meal	13.17	12.41	12.47	12.58
Rapeseed meal	4.00	4.00	4.00	0.00
Broad bean straw silage*	0.00	10.00	17.00	24.00
Soybean oil	0.00	0.00	1.42	2.90
Dicalcium phosphate	1.64	1.07	1.13	1.18
Stone powder	1.06	0.95	0.60	0.37
Premix	1.00	1.00	1.00	1.00
**Nutrition level**				
Metabolic energy (KC/Kg)	2,988	2,890	2,890	2,890
Crude protein (%)	14.10	14.00	14.00	14.00
Crude fiber (%)	2.40	4.20	5.50	6.80
Ca (%)	0.80	0.74	0.70	0.70
P (%)	0.50	0.50	0.50	0.50

Premix provided per kilogram of diet: V_A_ 13000 IU, V_D_ 33000 IU, V_E_ 35 mg, V_K_ 33 mg, V_B_ 12.5 mg, V_B_ 26 mg, V_B_ 63 mg, V_B_ 120.25 mg, Nicotinic acid 25 mg, Pantothenic acid 15 mg, Biotin 0.15 mg, Cu 150 mg, Fe 80 mg, Zn 80 mg, Mn 10 mg, I 0.3 mg, Se 0.2 mg. ^*^Nutrient and energy content of the silage: Dry matter (95 g/kg), Starch (11 g/kg), Energy (1890 KJ/kg), Crude protein (1.21%), Neutral detergent fiber (6.4%), Acid detergent fiber (5.2%), Ammoniacal nitrogen (380.74 mg/kg), Soluble sugar (0.65 g/kg).

### Extraction of Fecal Genomic DNA

Fecal DNA was extracted from the cecal and jejunal content samples using a QIAamp DNA Stool Mini Kit (Qiagen 51504) according to manufacturer's instructions. DNA purification was performed using QIAamp Mini Spin columns following the standard protocols. DNA concentration was determined using a NanoDrop ND-1000 (Thermo Scientific, Waltham, Massachusetts, USA).

### Amplification and Sequencing of 16S rRNA Genes

The 16S rRNA genes of 16S V3-V4 were amplified used specific primers (341F: 5′-CCTAYGGGRBGCASCAG-3′ and 806R 5′-GGACTACNNGGGTATCTAAT-3′) with the barcode. PCR was performed with 15 μl of Phusion® High-Fidelity PCR Master Mix (New England Biolabs). The PCR products were the detected by 2% agarose electrophoresis. Then, the PCR products were purified using the Qiagen Gel Extraction Kit (Qiagen, Germany).

Sequencing libraries were generated using the TruSeq® DNA PCR-Free Sample Preparation Kit (Illumina, USA) following the manufacturer's recommendations and index codes were added. The library quality was assessed on the Qubit 2.0 Fluorometer (Thermo Scientific) and the Agilent Bioanalyzer 2100 system. Finally, the library was sequenced on an Illumina NovaSeq platform and 250 bp paired-end reads were generated.

### Sequence Analysis

Paired-end reads were assigned to samples based on their unique barcode and were truncated by removing the barcode and primer sequence. Paired-end reads were merged using FLASH (V1.2.7, http://ccb.jhu.edu/software/FLASH/) (28). Quality filtering on the raw tags was performed under specific filtering conditions to obtain high-quality clean tags (29) according to the QIIME (V1.9.1, http://qiime.org/scripts/split_libraries_fastq.html) (30) quality control process. The tags were compared with a reference database (Silva database, https://www.arb-silva.de/) using the UCHIME algorithm (UCHIME Algorithm, https://www.drive5.com/usearch/manual/uchime_algo.html) (31) to detect chimera sequences, and the chimera sequences were then removed (32) to obtain effective tags.

Sequence analysis was performed using Uparse software (Uparse v7.0.1001, http://drive5.com/uparse/) (33). Sequences with ≥97% similarity were assigned to the same operational taxonomic units (OTUs). Representative sequence for each OTU was screened for further annotation. For each representative sequence, the Silva Database (http://www.arb-silva.de/) (34) was used based on Mothur algorithm to annotate taxonomic information. To study phylogenetic relationship of different OTUs, and the difference of the dominant species in different samples (groups), multiple sequence alignment were conducted using the MUSCLE software (Version 3.8.31, http://www.drive5.com/muscle/) (35). OTU abundance information was normalized using the standard sequence number corresponding to the sample with the least sequences. Subsequent analysis of α- and β-diversity were all performed based on these output normalized data.

We defined the top 200 abundant OTUs as core microbiota for α- and β-diversity analysis (36). Alpha diversity is applied for analyzing the complexity of species diversity for a sample through indices including the Shannon and Simpson indices. All indices in our samples were calculated using the MOTHUR program (http://www.mothur.org). β-diversity analysis was used to evaluate the differences in species complexity among samples. β-diversity on both weighted and unweighted UniFrac analyses were calculated using QIIME software (Version 1.9.1) (Version 2.15.3). Principal Coordinate Analysis (PCoA) was performed to obtain principal coordinates and were visualized from complex, multidimensional data using the vegan and ggplot2 packages in R-4.0.1. Dendrogram analyses were performed based on Bray Curtis distance using the vegan packages in R-4.0.1.

### LC-MS Metabolomics Processing

#### Metabolite Extraction

In total, 48 cecal and jejunal content samples were analyzed using the LC-MS platform (Thermo Fisher, Waltham, MA, USA). Tissues (100 mg) were individually ground in liquid nitrogen and the homogenate was resuspended in prechilled 80% methanol and 0.1% formic acid by vortexing. The samples were incubated on ice for 5 min and then centrifuged at 15,000 rpm, 4°C for 5 min. Some of the supernatant was diluted to a final concentration of 60% methanol with LC-MS grade water. The samples were subsequently transferred to a fresh microcentrifuge tube (Eppendorf) with a 0.22 μm filter and were then centrifuged at 15,000 rpm, 4°C for 10 min. Finally, the filtrate was used for LC-MS/MS analysis.

LC-MS/MS analyses were performed on a Vanquish UHPLC system (Thermo Fisher) coupled with an Orbitrap Q Exactive series mass spectrometer (Thermo Fisher). Samples were injected onto an Hyperil Gold column (100 × 2.1 mm, 1.9 μm) using a 16-min linear gradient at a flow rate of 0.2 ml/min. The eluents for the positive polarity mode were eluent A (0.1% formic acid in water) and eluent B (methanol). The eluents for the negative polarity mode were eluent A (5 mM ammonium acetate, pH 9.0) and eluent B (methanol). The solvent gradient was set as follows: 2% B, 1.5 min; 2–100% B, 12.0 min; 100% B, 14.0 min; 100–2% B, 14.1 min; 2% B, 16 min. The Q Exactive mass series spectrometer was operated in the positive/negative polarity mode with a spray voltage of 3.2 kV, capillary temperature of 320°C, sheath gas flow rate of 35 arb units and an aux gas flow rate of 10 arb units.

### Metabolomics Data Analysis

The raw data files generated by UHPLC-MS/MS were processed using Compound Discoverer 3.0 (CD3.0, Thermo Fisher) to perform peak alignment, peak picking, and quantitation for each metabolite. The main parameters were set as follows: retention time tolerance, 0.2 mins; actual mass tolerance, 5 ppm; signal intensity tolerance, 30%; signal/noise ratio, 3; and minimum intensity, 100,000. After this, peak intensities were normalized to the total spectral intensity. The normalized data were used to predict the molecular formula based on the additive ions, molecular ion peaks, and fragment ions. The peaks were then matched with the mzCloud (https://www.mzcloud.org/) and ChemSpider (http://www.chemspider.com/) databases to obtain accurate qualitative and relative quantitative results. Statistical analyses were performed using the statistical software R-4.0.1, Python (Python 2.7.6 version), and CentOS (CentOS release 6.6), When data were not normally distributed, normal transformations were attempted using an area normalization method.

These metabolites were annotated using the KEGG (http://www.genome.jp/kegg/), HMDB (http://www.hmdb.ca/), and Lipidmaps (http://www.lipidmaps.org/) databases. Orthogonal partial least-squares discriminant analysis (OPLS-DA) was performed with metaX (a flexible and comprehensive software for processing metabolomics data) to discriminate the metabolic profiles across groups. We applied univariate analysis (t-test) to calculate the statistical significance (*p*-value). The metabolites with variable importance in the projection (VIP) > 1 and *p* < 0.05 and fold change (FC) ≥ 2 or ≤ 0.5 were considered differential metabolites. The impact of the gut segment and DF on the metabolic pathways by metabolite set enrichment analysis was performed using an online tool [http://www.metaboanalyst.ca/MetaboAnalyst/faces/ModuleView.xhtml (37)].

For clustering heat maps, the data were normalized using the z-scores of intensity areas of differential metabolites and were plotted with the “Pheatmap” package in R-4.0.1. The correlations between differential metabolites were analyzed using Spearman's rank correlation in R-4.0.1. The functions of these metabolites and metabolic pathways were studied using the KEGG database.

### Statistical Analysis

The software R-4.0.1 was used for statistical analyses. To investigate the effect of gastrointestinal tract and DF on the core bacteria in the gut of Durco × Bamei crossbred pigs, we used the top 200 abundant OTUs for α-and β-diversity analysis. Significance for α-diversity was detected using the Wilcoxon test. PCoA were based on weighted and unweighted UniFrac distances, and significance was checked with multivariate permutation tests using the non-parametric method “ADONIS” included in the package “vegan.” The linear discriminant analysis (LDA) Effect Size (LEfSe) method was used to assess differences in microbial communities among different groups. Spearman correlations were used to calculate the correlation between bacterial genera and metabolites.

## Results

### Differences in Gut Bacterial Community Composition and Diversity Between Two Gut Segments From Durco × Bamei Crossbred Pigs

**Figure 1:**
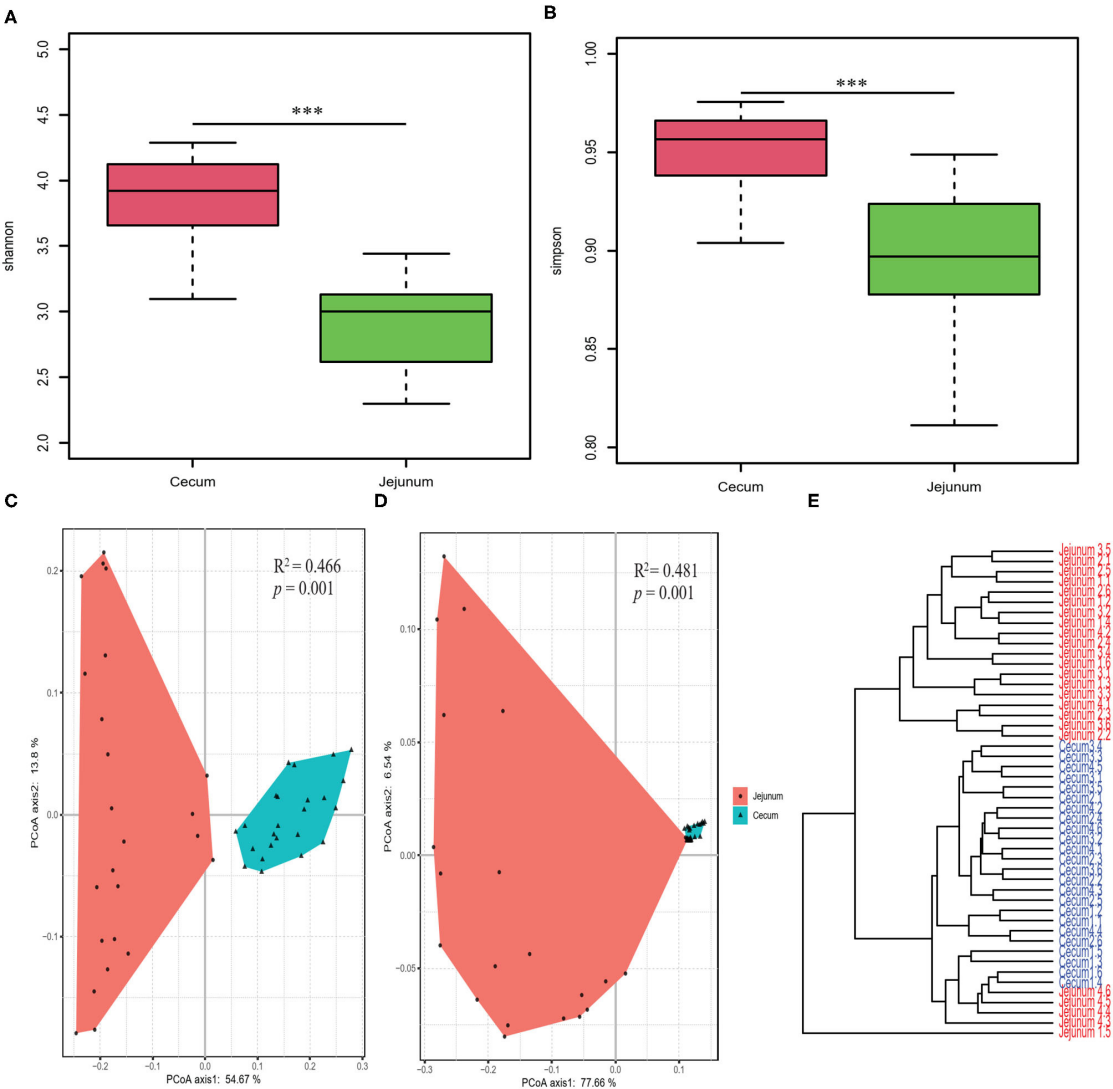
Diversity analysis of core bacteria between two gut segments. The α- and β-diversity comparison for the cecum and jejunum. **(A,B)** Box plots showing significantly different α-diversity between the jejunal and cecal content samples by Wilcoxon test. PCoA based on weighted **(C)** and unweighted **(D)** UniFrac distances. Adonis tests show statistically significant differences between the cecum and jejunum. **(E)** Dendrogram analyses were performed based on the Bray Curtis distance. *p* values: ***p < 0.001.

We performed OTU analysis and compared the α-diversity of the microbiota from the two intestinal regions. Both the Simpson and Shannon indices were significantly higher in cecal bacteria compared to those in the jejunum (Wilcoxon test, all *p* < 0.001; Figures 1A,B). PCoA based on weighted and unweighted UniFrac distance matrices, showed that the bacterial composition differed significantly between the two gut segments (Figures 1C,D). Furthermore, Bray Curtis clustering analysis of gut bacteria at the OTU level showed that most samples from the two gut segments could be clustered into two subgroups (Figure 1E).

**Figure 2:**
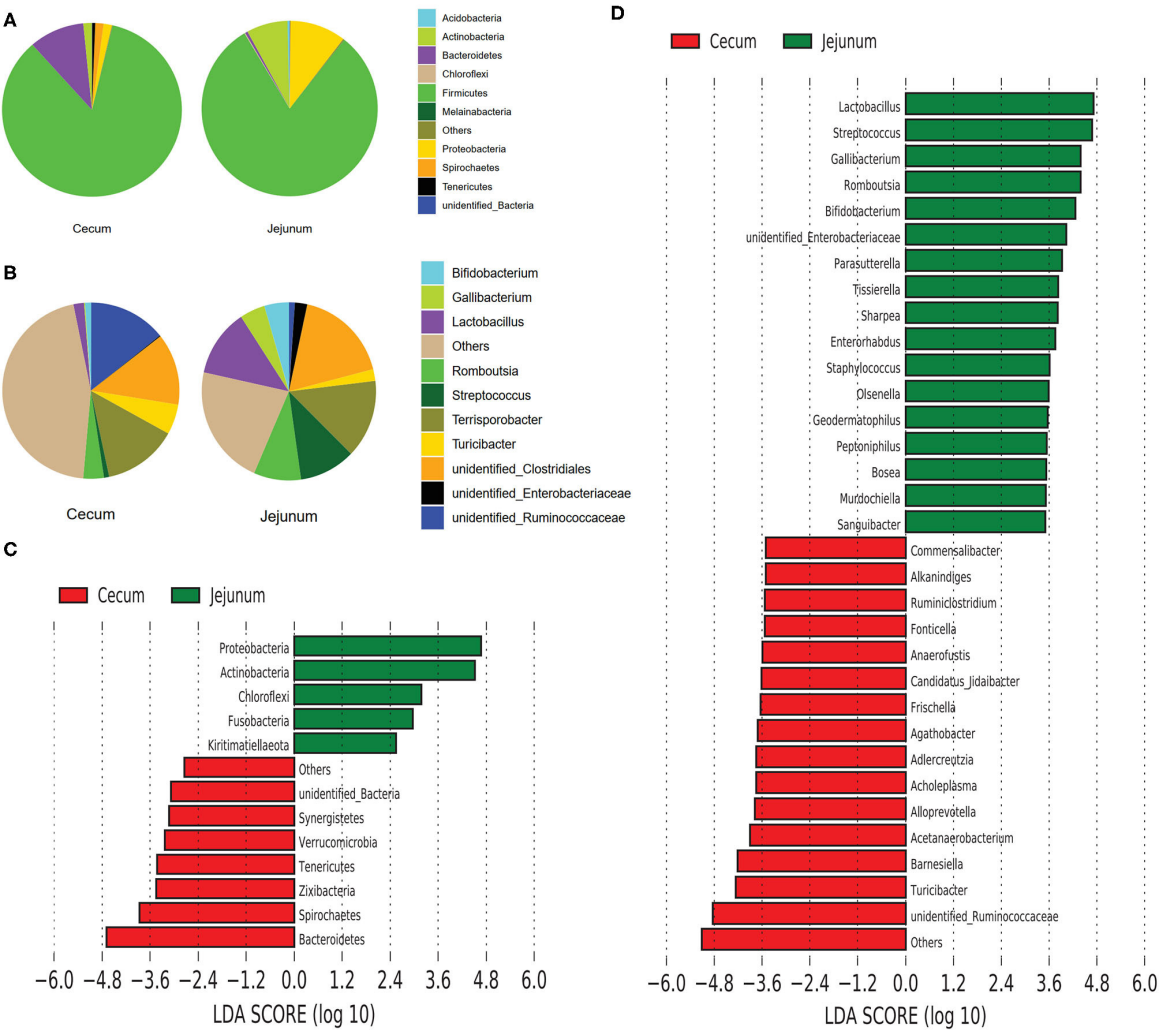
Composition analysis of bacteria from the two gut segments. **(A)** Microbial composition at the phylum **(A)** and genus **(B)** level. LEfSe identified significantly different bacteria according to the relative abundance between the two gut segments at the phylum level **(C)** and genus level **(D)** (*p* < 0.05, LDA Score > ± 2.0 at the phylum level, and LDA Score > ± 3.5 at the genus level).

We explored the taxonomic distribution of numerically abundant bacteria in each gut segment. Among the top 10 abundant phyla, Firmicutes constituted the most prevalent phylotype in the two gut segments comprising 84.76% of the relative abundances in the cecal bacterial population 80.76% of that in the jejunal bacterial population, followed by Bacteroidetes occupied 9.97% of the bacterial population in the cecum, and Proteobacteria occupied 10.29% of the bacterial population in the jejunum (Figure 2A). However, Bacteroidetes only accounted for 0.5% in the jejunum, and Proteobacteria only accounted for 1.51% in the cecum. The relative abundances of Actinobacteria were 1.54 and 7.58% in the cecum and jejunum, respectively (Figure 2A). Among the 10 most abundant genera, the pie chart showed that the total abundance of the top 10 genera only accounted for 54.55% of the entire bacterial population in the cecum; however, they accounted for 78.1% of the bacterial population in the jejunum (Figure 2B). Unidentified *Ruminococcaceae* (14.24%), *Terrisporobacter* (13.6%), and unidentified *Clostridiales* (13.17%) were the most prevalent genera in the cecum, whereas Unidentified *Clostridiales* (17.59%), *Terrisporobacter* (14.23%), *Lactobacillus* (12.34%), *Streptococcus* (10.4%), and *Romboutsia* (8.79%) were the five most prevalent genera in the jejunum (Figure 2B).

We performed linear discriminant analysis coupled with effect size (LEfSe) on taxa that exhibited linear discriminant analysis (LDA) scores greater than 2.5 at the phylum level and 3.5 at the genus level. The results indicated 12 phyla that were differentially represented between the two gut segments (Figure 2C). These phyla included Proteobacteria, Actinobacteria, Chloroflexi, Fusobacteria, Euryarchaeota, and Kirtimatiellaeota, which were enriched in the jejunum, and unidentified Bacteria, Synergistetes, Verrucomicrobia, Tenericutes, Zixibacteria, Spirochaetes, and Bacteroidetes, which were relatively more abundant in the cecum. In all, 13 genera including *Lactobacillus, Streptococcus, Gallibacterium, Romboutsia*, and *Bifidobacterium* were relatively more abundant in the jejunum than in the cecum, whereas 15 genera including *Commensalibacter, Alkanindiges, Ruminiclostridium, Turicibacter*, and unidentified *Ruminococcaceae* were relatively more abundant in the cecum than in the jejunum (Figure 2D).

### Comparison of Microbial Community Diversity in Different Gut Segments of Durco × Bamei Crossbred Pigs From the Four Dietary Groups

**Figure 3:**
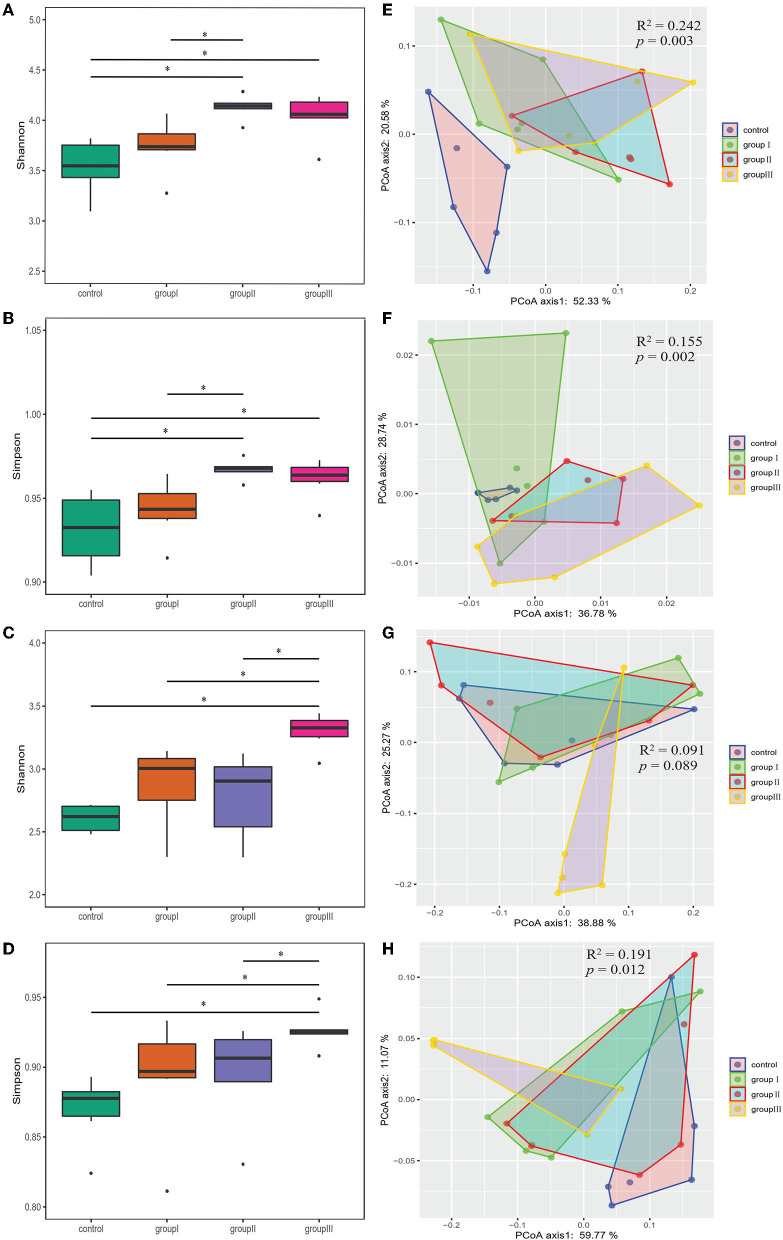
Comparing α- and β-diversity of core bacteria from four dietary groups in two gut segments. Box plots showing significantly different Shannon **(A)** and Simpson **(B)** diversity indices among four dietary groups in the cecum **(A,B)** and jejunum **(C,D)** by Wilcoxon test. PCoA based on weighted **(E,G)** and unweighted **(F,H)** UniFrac distances for the cecum **(E,F)** and jejunum **(G,H)** bacterial populations. Adonis tests showed statistically significant differences among four dietary groups. *p < 0.05.

Both Shannon and Simpson indices were significantly higher in group II and group III compared with those in the control group in the cecum (Wilcoxon test, *p* < 0.05; Figures 3A,B). Group III showed significantly higher Shannon and Simpson indices than the control group, group I and II in the jejunum (Wilcoxon test, *p* < 0.05; Figures 3C,D). The results of PCoA based on weighted UniFrac and unweighted UniFrac distance showed that the bacterial community from the control group was obviously separated from group I, group II, and group III in the cecum (Adonis analysis, all *p* < 0.05; Figures 3E,F). However, the influence of DF on bacterial community structure in jejunum was weak compared to that in cecum (Figures 3G,H).

As the results showed that both gastrointestinal tract and DF significantly affected the bacterial community diversity, we performed two-way ANOVA analysis to found out which factor contribute more to the variation of bacterial diversity, and whether there is an interaction effect between gastrointestinal tract and DF. The results indicated gastrointestinal tract had more relative contributions impacting bacterial diversity than DF, and their interaction had significant impact on Shannon index (Supplementary Table 1).

### Comparison of Microbial Community Compositions in the Cecum of Durco × Bamei Crossbred Pigs From Different Dietary Groups

**Figure 4:**
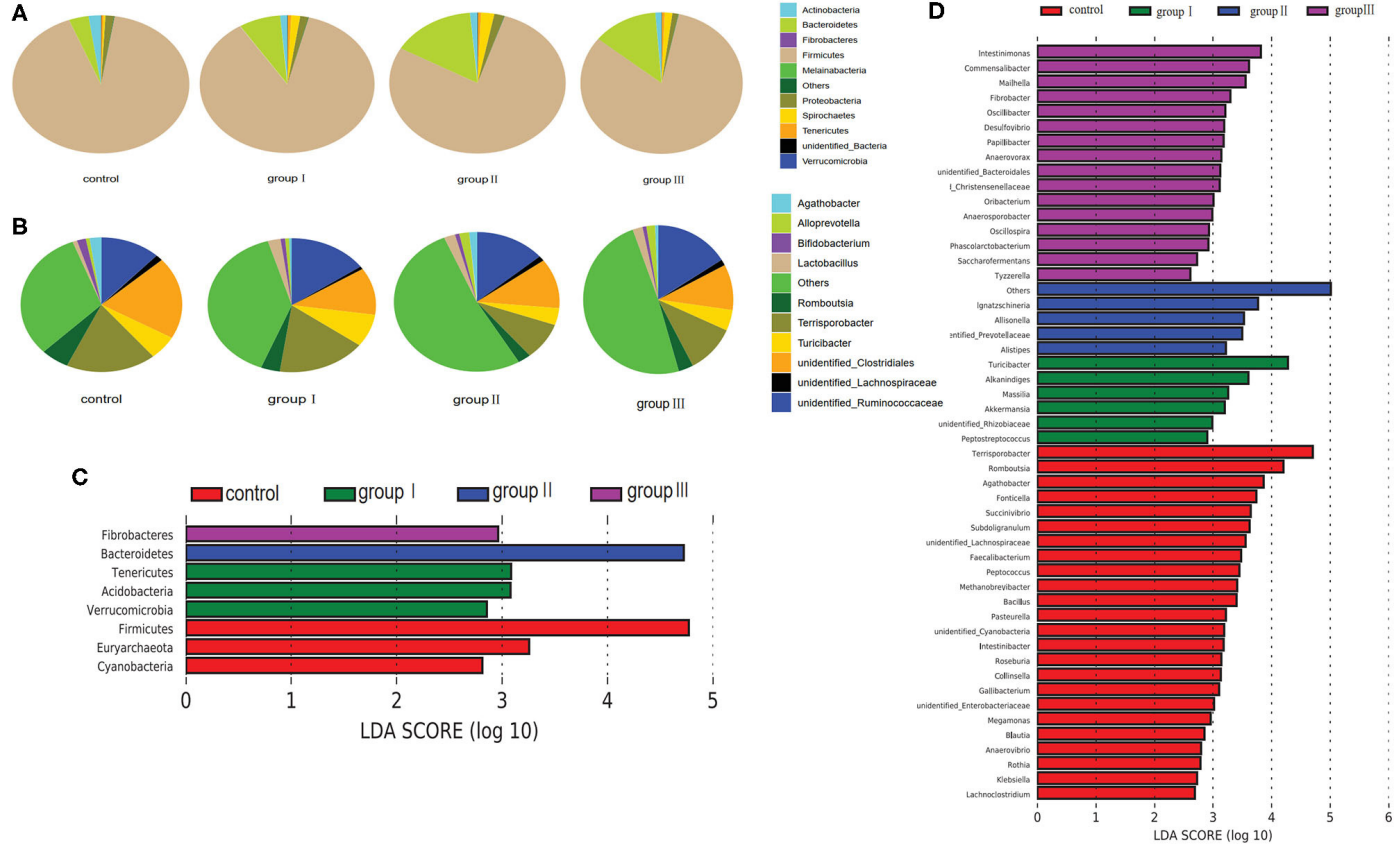
Composition analysis of bacteria in the cecum from four dietary groups. Microbial composition at the phylum **(A)** and genus **(B)** level. LEfSe identified significantly different bacteria according to relative abundance among the four dietary groups at the phylum **(C)** and genus level **(D)** (*p* < 0.05, LDA Score > ± 2.0 at the phylum level, and LDA Score > ± 2.5 at the genus level).

Based on the top 10 abundant bacterial phyla in cecal samples, Firmicutes constituted the most prevalent phylotype in all four groups comprising 85% of the control group microbial population, 79.3, 70.3, and 74.4% of that in group I, group II, and group III, respectively, followed by Bacteroidetes, which occupied 3.6, 7.6, 15, and 12.9% of the control, group I, group II, and group III microbial population, respectively (Figure 4A). Actinobacteria accounted for 2.3% of the microbial population in the control group, but only accounted for 1.3, 1.4, and 1.2% of that in group I, group II and group III, respectively (Figure 4A). Improved proportions of DF contributed to an increase in the relative abundance of Spirochaetes which was 1.6, 2.4, and 1.5% in group I, group II, and group III, respectively, but only accounted for 0.4% of the microbial population in the control group (Figure 4A). At the genus level, unidentified *Clostridiales* (19.3%) were the most prevalent in the control group, but were reduced in group I (11.1%), group II (11.3%), and group III (9.7%) (Figure 4B). The relative abundance of *Terrisporobacter* and *Romboutsia* in the control group were 18.1 and 5.7%, whereas they reduced in group I (17.1, 3.7%), group II (8.6, 2.6%), and group III (10.6, 3.2%) (Figure 4B). However, the relative abundance of unidentified *Ruminococcaceae* was increased in group I (9.7%), group II (8.6%), and group III (11%), compared with the control group (7.7%). While group II had higher abundance of *Turicibacter* (7.9%) compared with the control group (5.7%), group II (3.9%), and group III (4.5%) (Figure 4B).

To identify specific bacterial species that were characteristic to the four dietary groups in the cecum, we performed LDA (Figures 4C,D). Fibrobacteres and Bacteroidetes were enriched in group III and group II respectively. Tenericutes, Acidobacteria, and Verrucomicrobia were enriched in group I, whereas Firmicutes, Euryarchaeota, and Cyanobacteria enriched in the control group. At the genus level, 51 genera were differentially represented among the four dietary groups (Figure 4D). Of these, 16 bacterial genera including two fiber-degrading bacteria, *Fibrobacter* and unidentified *Prevotellaceae*, were enriched in group III; 4 genera including *Ignatzschineria, Allisonella, Alistipes*, and unidentified *Prevotellaceae* were enriched in group II; 6 genera including 1 fiber-degrading bacteria *Akkermansia*, were displayed more abundantly in group II. However, 24 genera, including 1 bile salt hydrolase-related bacteria *Bacillus* enriched in control group.

### Comparison of Microbial Community Composition in the Jejunum of Durco × Bamei Crossbred Pigs From Four Dietary Groups

**Figure 5:**
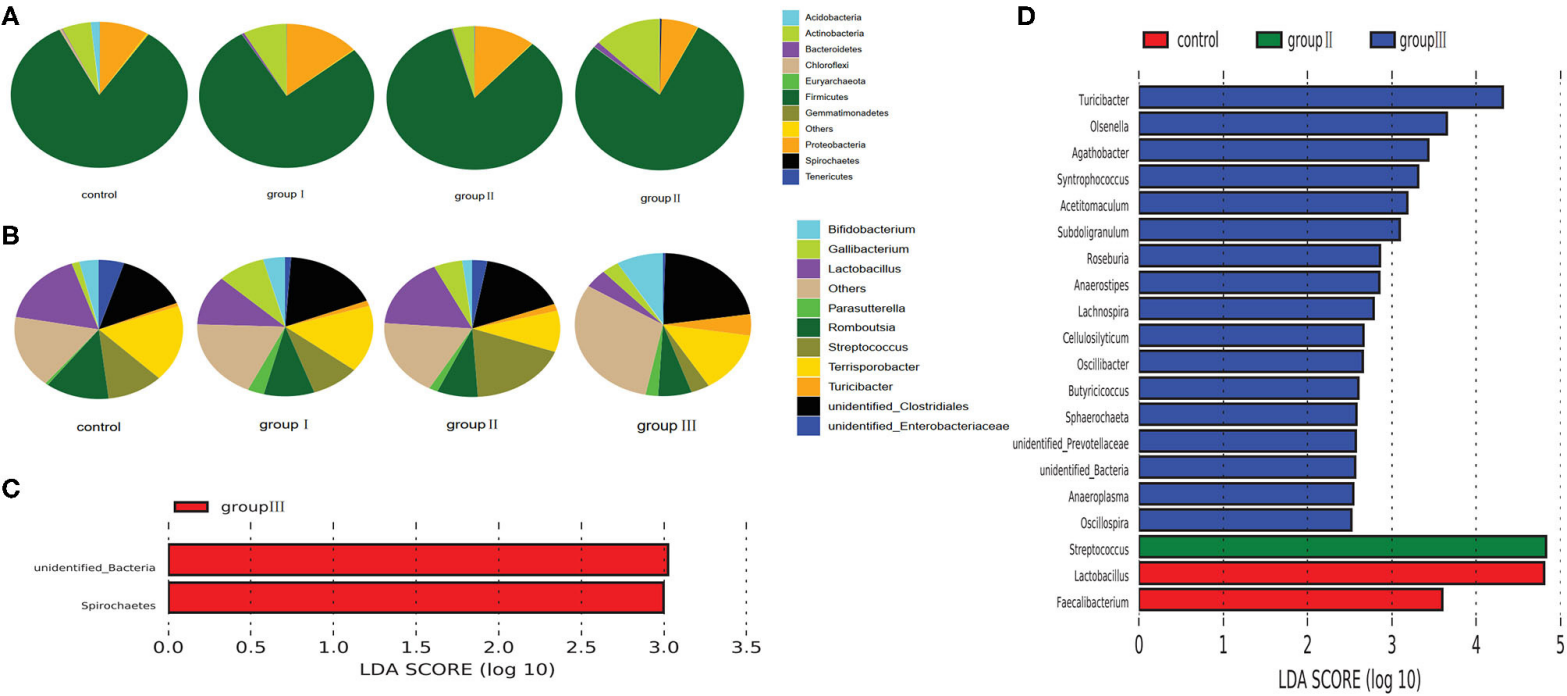
Composition analysis of bacteria in the jejunum from four dietary groups. Microbial composition at the phylum **(A)** and genus **(B)** level. LEfSe identified significantly different bacteria according to the relative abundance among the four dietary groups at the phylum level **(C)** and genus level **(D)** (*p* < 0.05, LDA Score > ± 2.0 at the phylum level, and LDA Score > ± 2.5 at the genus level).

Based on the top 10 abundant bacterial phyla in jejunum samples, Firmicutes constituted the most prevalent phylotype in all four groups, comprising 83.13% of the microbial population in the control group, and 77.36, 84.3, and 78.27% of that in group I, group II and group III, respectively, followed by Proteobacteria, which occupied 8.7, 13.8, 11.1% of the control, group I, and group II, whereas Actinobacteria was the second prevalent phylotype in group III, comprising 12.9% of the microbial population (Figure 5A). The third prevalent phylotype in the control group, group I, and group II was Actinobacteria, comprising 5.3%, 8.1%, and 4% of the microbial population, respectively. Proteobacteria was the third prevalent phylotype in group III, which accounted 7.1% of the microbial population (Figure 5A). At the genus level, *Terrisporobacter* (17.77%) was the most prevalent in the control group, but the relative abundance of *Terrisporobacter* was reduced in group I (15.56%), group II (9.74%), and group III (13.86%). The relative abundance of *Lactobacillus, Romboutsia*, and unidentified *Enterobacteriaceae* in the control group were 16.9, 12.22, and 4.87%, respectively, whereas it was reduced in group I (11.16, 9.29, and 1.07%), group II (16.62, 7.51, and 2.76%), and group III (4.21, 6.14, and 0.45%) with an increase in DF. However, the relative abundance of unidentified *Clostridiales* was increased in group I (17.82%), group II (16.39%), and group III (22.2%), compared with that in the control group (13.93%). The relative abundance of *Turicibacter* was also increased with the increase in dietary fiber, was accounting for 0.78, 1.25, 1.65, and 4.8% in the control, group I, group II, and group III, respectively (Figure 5B).

To identify specific bacterial species characteristic to the four dietary groups in the jejunum, we performed LDA. At the phylum level, the relative abundance of unidentified Bacteria and Spirochaetes was higher in group III than that in the control, group I, and group II (Figure 5C). At the genus level, 20 genera were differentially represented among the four dietary groups (Figure 5D). Of these, 17 bacterial genera were enriched in group III, *Streptococcus* was more abundant in group II than in the other three dietary groups; and two genera including *Lactobacillus* and *Faecalibacterium* were enriched in the control group.

### Metabolic Profiling of Gut Microbes in the Cecum and Jejunum of Durco × Bamei Crossbred Pigs

**Figure 6:**
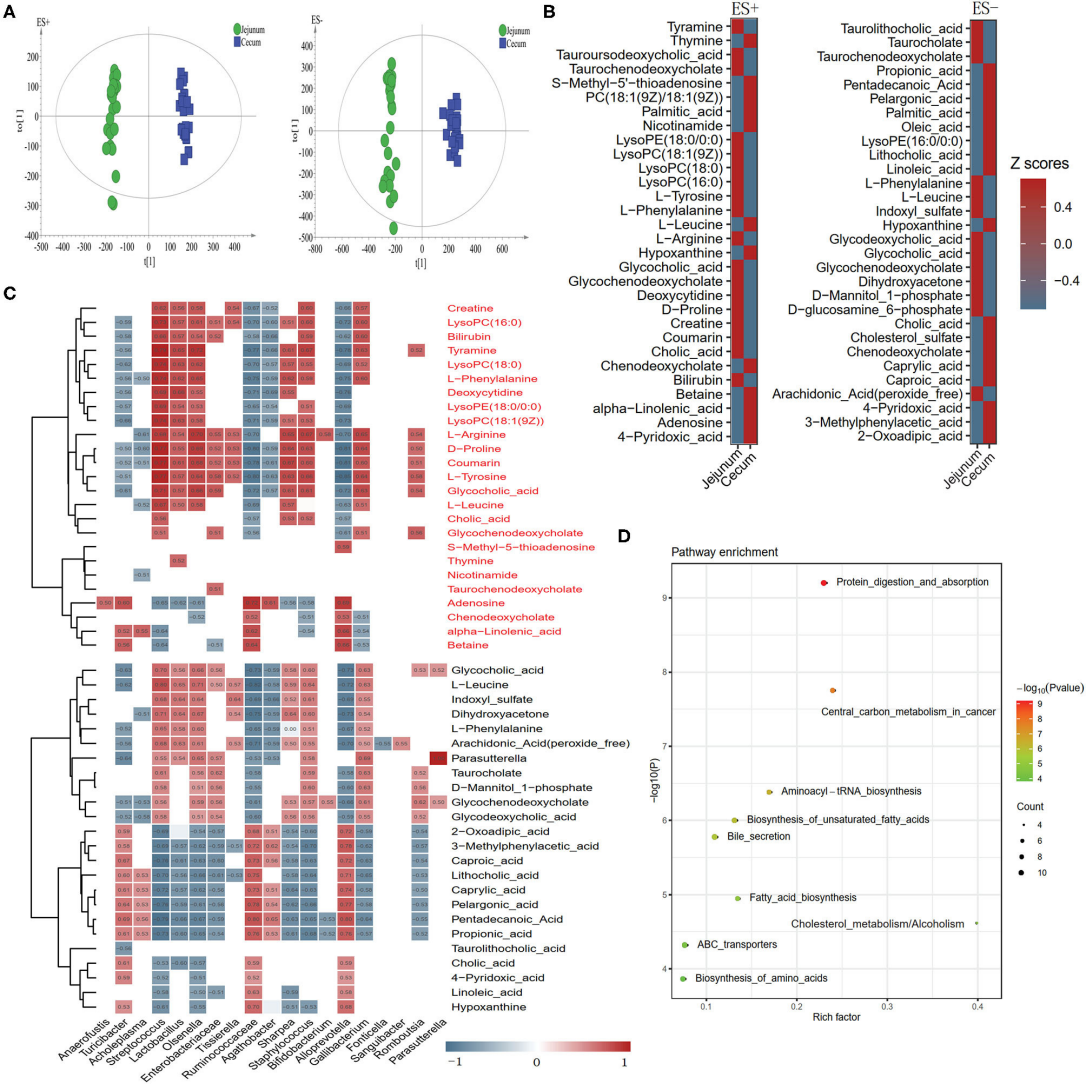
Different metabolic patterns in the cecum and jejunum. **(A)** Score scatter plots of OPLS-DA based on the metabolic profiles in content samples from the cecum and jejunum of pigs in ES+ and ES–. **(B)** The top 30 metabolites in ES+ and ES– that were significantly changed in the cecum compared to the jejunum at VIP > 1 and *p* value (t test) < 0.05 are identified. **(C)** The relationship between 60 endogenous metabolites and the top 30 altered genera (Figure 2D) in the cecum and jejunum is estimated by Spearman's correlation analysis. Those with low correlation (|*r*| < 0.5) are not shown. **(D)** The top 10 pathway enrichment analyses performed using the significantly different metabolites between the cecum and jejunum.

We explored the metabolic profile of microbes in the cecum and jejunum of the 24 pigs using high-throughput LC/MS. Samples from cecal content was separated from the jejunum according to the OPLS-DA score scatter plots (Figure 6A). The compositional changes in different gut segments involved 131 analytes that were significantly different between the cecum and jejunum. Among the top 30 richness altered metabolites both in ES+ and ES–, fatty acids including palmitic acid, alpha-linolenic acid, and free bile acids including cholic acid and chenodeoxycholate were enriched in the cecum (Figure 6B). In contrast, conjected bile acids including taurolithocholic acid, glycodeoxycholic acid and glycocholic acid, and amino acids including L-phenylalanine, L-leucine and L-tyrosine were enriched in the jejunum (Figure 6B). Cecum-enriched metabolites were mainly positively linked with unidentified *Ruminococcaceae, Turicibacte, Acholeplasma, Agathobacter, Alloprevotella*, and *Streptococcus* (Figure 6C). Jejunum-enriched metabolites were mainly positively linked to *Streptococcus, Lactobacillus, Olsenella*, unidentified *Enterobacteriaceae, Agathobacter, Sharpea, Gallibacterium*, and *Romboutsia* (Figure 6C). Of these, altered metabolites were the most enriched in pathways including protein digestion and absorption, central carbon metabolism in cancer, aminoacyl—tRNA biosynthesis, biosynthesis of unsaturated fatty acids, and bile secretion (Figure 6D).

### Metabolic Profiling of Gut Microbes in the Cecum of Durco × Bamei Crossbred Pigs From Four Dietary Groups

**Figure 7:**
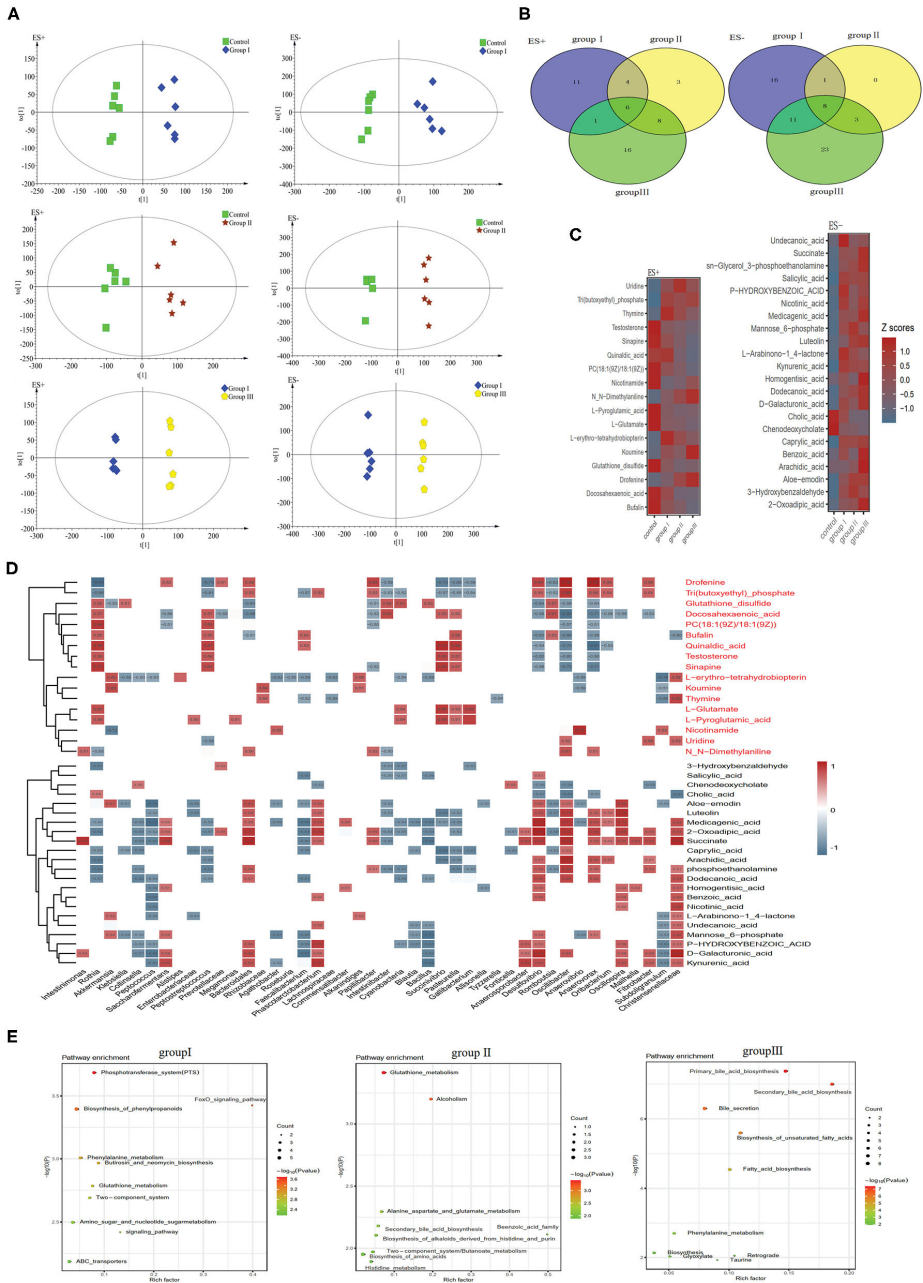
Different metabolic patterns in group I, group II, and group III. **(A)** Score scatter plots of OPLS-DA comparing the metabolic differences identify the separation between group I and control, group II and control, and group III and control, respectively. **(B)** Metabolites that are significantly changed in group I, group II, or group III compared to the control at VIP > 1 and *p* value (t test) < 0.05 are identified. Venn diagrams demonstrate the number of altered metabolites shared among group I (blue), group II (yellow) and group III (green) by the overlap. **(C)** The relative amounts of 42 metabolites that varied concurrently among group I, group II, and group III are transformed into Z scores in the heat map. **(D)** The relationship between 42 metabolites and the 30 top altered genera (Figure 4D) in group I, group II, and group III was estimated by Spearman's correlation analysis. Those with low correlation (|*r*| < 0.5) are not shown. **(E)** The top ten pathway enrichment analyses performed using significantly different metabolites between the control group and three treatment groups.

To discriminate metabolic profiles across groups, we performed clustering analyses based on OPLS-DA. Samples from group I, group II, and group III were all separated from the control group according to the OPLS-DA score scatter plots (Figure 7A). The compositional changes in individuals from the treatment involved 167 analytes that were significantly different between the treatment groups and the control and 58, 33, and 76 analytes were altered in group I, group II, and group III, respectively (Figure 7B). There were 10 metabolites that were obviously different in all three treatment groups compared to the control group (Figure 7C). Metabolites whose levels were significantly decreased with the DF levels include chenodeoxycholate, L-glutamate, and L-pyroglutamic acid, whereas metabolites whose levels significantly increased along the dietary levels including 2-oxoadipic acid, caprylic acid, medicagenic acid, sn-glycerol 3-phosphoethanolamine, and succinate (Figure 7C). Additionally, carbohydrates including mannose 6-phosphate and D-galacturonic acid, and fatty acids including undecanoic acid, dodecanoic acid, and arachidic acid were enriched in the high DF groups (Figure 7C). Control-enriched metabolites were positively linked to *Rothia, Cyanobacteria, Succinivibrio, Pasteurella*, and *Gallibacterium*, whereas treatment-enriched metabolites were positively linked to *Saccharofermentans, Bacteroidales, Phascolarctobacterium, Anaerosporobacter, Desulfovibrio, Oscillibacter, Anaerovorax, Oscillospira*, and *Christensenellaceae* (Figure 7D). Carbohydrates were positively linked to *Saccharofermentans, Desulfovibrio, Christensenellaceae*, whereas fatty acids were positively linked to *Desulfovibrio, Oscillibacter*, and *Anaerovorax* (Figure 7D). Of these altered metabolites, the most were enriched in the pathways including phosphotransferase system (PTS), amino acid metabolism, primary bile acid biosynthesis, secondary bile acid biosynthesis, and fatty acid biosynthesis (Figure 7D).

## Discussion

In this study, our results showed that the α-diversity and richness of the bacterial community in the cecum were significantly higher than those in the jejunum (Figures 1A,B), and the PCoA based on weighted and unweighted UniFrac distance showed that the cecal bacteria were obviously separated from the jejunal bacterial community suggesting that the β-diversity also differs significantly between the two gut segments (Figures 1C,D). These results were consistent with the finding in Jinhua, Landrace and Laiwu pigs which also showed that the α- and β-diversity of the bacterial community were significantly different between the two gut segments (5, 38). The variation of bacterial community diversity in different gut segments was also found in other livestock including dairy cattle, commercial pigs, goats, and chicken (4, 5, 39, 40), suggesting that the spatial heterogeneity in the composition and diversity of gut bacteria was ubiquitous among livestock. The spatial heterogeneity of the gut bacterial community could be explained by the distinct composition and structure of epithelial cells in different gut segments, resulting in differences in gut motility, digestive enzymes and pH (2, 4, 41). The high diversity of the bacterial community in cecum was ubiquitous in pigs of different breeds (e.g., Duroc, Jinhua, Landrace, Laiwu, Large White) (5, 38, 42). This could be attributed to the fact that the cecum is the main gut segment for gut bacteria to ferment DF, which contributes to cecum bacterial reproduction, resulting in higher richness of bacterial taxa (43).

The spatial heterogeneity was also reflected in the composition of the predominant taxa (Figures 2A,B). Our results showed that the Firmicutes and Bacteroidetes were the 2 predominant bacterial phyla in the cecum, whereas Firmicutes, Proteobacteria, and Actinobacteria were the top 3 dominant phyla in the jejunum of Durco × Bamei crossbred pigs (Figure 2A). Consistent with previous findings, the predominant phyla in cecum were shared with Jinhua and Landrace pigs, but the predominant phyla in the jejunum were different among the three breeds (38). The consistency of predominant bacterial phyla in the cecum among different breeds could be attributed to the similar diet-driven convergence of gut microbiomes possessing similar functions in the cecum (44), while the variation of predominant bacterial phyla in the jejunum may attribute to the secretions in small were distinction among different breeds (45). Furthermore, the predominated bacteria phyla in the cecum of Durco × Bamei crossbred pigs were also consistent with the findings in the cecum of chicken, rumen of goat, dairy cattle, and elk (*Cervus canadensis*) (4, 7, 39, 46), indicating that these bacteria capable of digesting DF were consistent among livestock.

Adding fiber to the chow of Duroc × Bamei crossbred pigs significantly altered the α-and β-diversity of bacterial community in the cecum and jejunum (Figure 3). These results can be explained by the fact that DF increases the reproduction and growth of bacteria capable of fermenting DF, resulting in variation in bacterial community diversity (10). Additionally, gut bacterial community reached the highest diversity in the cecum of group II, whereas it was reduced with increasing DF (Figures 3A,B). A similar result was also found in Suihuai pigs, which showed that individuals fed chow with 14% DF had the highest α-diversity, whereas the diversity indices decreased with increasing proportions of DF (12).

The relative abundance of Firmicutes in the cecum was decreased with the increase in DF, which is contrary to the findings with Duroc × (Landrace × Yorkshire) crossbred piglets (10). This difference could be derived from three aspects. First, the predominant bacterial taxa are distinct among different ages of the experimental individuals (40), which could result in distinct responses to DF; second, different sources of DF could lead to the amplification of different bacteria (43); third, the segments of sample collection can also influence bacterial composition significantly (47). The increased Bacteroidetes in high DF groups could be partly attributed to the increase in *Alloprevotella*, which produces acetic acid and succinic acid by fermenting carbohydrates (48). Our results also showed that the cellulose-degrading bacteria, Fibrobacteres, were enriched in the high dietary fiber groups (49). Although the richness of predominant bacterial phyla and genera in the jejunum fluctuated among the four groups, only a few of them were significantly different (Figures 5A–D). Of these significantly altered taxa, *Olsenella* has been reported as the lactic acid bacteria found in the jejunum of sheep and pigs (50). Altogether, our results implied that the variation in bacterial composition between the two gut segments is distinct in the response to DF.

Compared with bacterial metabolites in the jejunum, free bile acids were enriched in the cecum (Figure 6B). This result could be attributed to the richness of bacterial genera including *Lactobacillus, Streptococcus*, and *Bifidobacterium* which are capable of deconjugating conjugated bile acids to form free bile acids that can escape uptake in the small intestine and enter the cecum (45). Several upregulated fatty acids in the cecum are positively linked to unidentified *Ruminococcaceae* and *Agathobacter* (Figure 6C), which belong to Clostridiales. A previous study on rumen bacteria confirmed that many bacteria taxa belonging to Clostridiales can produce conjugated fatty acids (51), indicating that unidentified *Ruminococcaceae* and *Agathobacter* maybe critical bacteria genera involved in fatty acids synthesis. Additionally, KEGG analysis also confirmed that the distinct function of bacteria between the two gut segments were mainly involved in amino acid, fatty acid, and bile acid metabolism (Figure 6D).

Compared with the cecal metabolites, we detected higher concentrations of conjugated bile acids in jejunal metabolites (Figure 6B). This could be attributed to the processes of bile acid metabolism occurring in the small intestine (45). Additionally, many kinds of amino acids (e.g., L-phenylalanine, L-tyrosine,L-leucine, and D-proline) were enriched in the jejunum (Figure 6B). Dietary protein is usually converted into amino acids in the small intestine by both host and bacterial proteases (52). A previous study suggested that *Bifidobacterium, Lactobacillus*, and *Streptococcus* can produce proteases and peptidases that can assist host in dietary protein digestion (52). Moreover, correlation analysis showed that the enriched amino acids were positively linked to *Bifidobacterium, Lactobacillus*, and *Streptococcus* which was enriched in the jejunum (Figure 6C). These findings implied that *Streptococcus* was potentially beneficial in helping Durco × Bamei crossbred pigs to digest dietary protein and synthesize amino acids.

DF significantly altered the concentrations of several cecal metabolites. In total, 59, 34, and 78 metabolites were detected to be significantly different between the control group and group I, group II, and group III, respectively (Figure 7B). Among these metabolites, 2-oxoadipic acid and succinate were enriched in all treatments (Figure 7C). A previous study showed that 2-oxoadipic acid was a common metabolite of tryptophan and lysine, indicating that amino acid metabolism was active in high-DF groups (53). Succinate is an important intermediate of the tricarboxylic acid (TCA) cycle. The TCA cycle is the final common oxidative pathway for carbohydrates, fats, and amino acids, and is the most important metabolic pathway for energy and nutrition supply to the host (54). The high concentration of succinate in all three treatment groups may indicate that DF increased the energy metabolism of host gut bacteria in Durco × Bamei crossbred pigs. Similar results were found in Suhuai pigs and weaned piglets, indicating that adding appropriate fiber to diet promoted energy metabolism and improved growth performance in the host (10, 12). The concentration of two carbohydrates and carbohydrate conjugate metabolites, mannose 6-phosphate and D-galacturonic acid, were significantly higher in two treatment groups compared with those in the control group. Mannose 6-phosphate can be converted to fructose 6-phosphate through interaction with mannose-6-phosphate isomerase (55), and D-galacturonic acid is the main constituent of pectin (56), which may indicate that host activity in DF metabolism. Four fatty acids, caprylic acid, arachidic acid, dodecanoic acid, and undecanoic acid, were upregulated in the two high DF groups. This can be attributed to the influence of increased DF on bile acid metabolism, which may reduce the host capability of dietary lipid digestion and fatty acid absorption (57, 58), resulting in higher concentration of fatty acids in the cecum. Similar results were also found in Duroc × Landrace × Large White pigs fed with a highly resistant starch diet (47).

The concentration of several organic acids such as benzoic acid, homogentisic acid, kynurenic acid, salicylic acid, and nicotinic acid also enhanced in the high DF groups (Figure 7C). Homogentisic acid is an intermediate in the breakdown or tyrosine and phenylalanine, indicating that tyrosine and phenylalanine metabolism was active in high DF groups (59). Nicotinic acid is a water-soluble vitamin whose derivatives, NADH, NAD, NAD+, and NADP, play essential roles in energy metabolism (60). Additionally, we observed alterations in the concentrations of two purine metabolites, thymine and uridine (Figure 7C). Dietary nitrogen from food resources is degraded and reused by the bacterial community for nucleic acid synthesis, which may explain the high bacterial richness in high DF groups (61, 62). Furthermore, KEGG analysis showed that altered metabolites were enriched in functional pathways including carbohydrate metabolism, lipid metabolism, amino acid metabolism, and digestive system (Figure 7D), indicating that DF significantly altered the energy and nutrition metabolism of Durco × Bamei crossbred pigs.

Among these altered metabolites and bacterial genera, we found a strong positive correlation between cecal bacterial metabolites and genera. The 2-oxoadipic acid was positively linked with *Clostridia, Fibrobacteria* and *Bacteroidales* (Figure 7D). *Oscillibacter* was positively correlated with many fatty acids including caprylic acid, arachidic acid, and dodecanoic acid in our results. Similar results were also found in a study on bovine ruminal metabolites, which showed that the bacterial genus *Oscillibacter* was closely related with the increased fatty acids in the high forage/concentrate diet group (63). Additionally, Wu et al. (64) also found that the relative abundance of *Oscillibacter* was positively correlated with medium chain fatty acid production in a fermentor for fermenting domestic wastewater. The results showed that the concentration of succinate was positively linked with many bacterial genera belonging to Clostridia (Figure 7D). Studies on the human gut microbiome and metabolism indicated that *Clostridium* is capable of converting succinate into SCFAs (65). Additionally, unidentified *Bacteroidales*, which are capable of fermenting dietary carbohydrates and proteins into succinate in the human gut (65), were positively linked with succinate. The bacterial genera *Oribacterium, Anaerosporobacter* and *Papillibacter*, unidentified Christensenellaceae, *Intestinimonas, Saccharofermentans, Oscillibacter, Anaerovorax*, and *Oscillospira* which belong to Clostridia, were also positively linked with succinate (Figure 7D), indicating that these bacterial genera may be capable of fermenting DF in the cecum of Durco × Bamei crossbred pigs. Mannose 6-phosphate and D-galacturonic acid were also positively linked with Clostridia (Figure 7D). Previous studies on the human and mouse gut microbiota suggested that DF significantly decreased bile acids, because of a decrease in genera including *Lactobacillus, Bacillus, Streptococcus*, and *Lactococcus* (66, 67). The common function of these reduced bacterial genera is to generate bile salt hydrolase enzymes that are capable of deconjugating conjugated bile acids (66, 67).

## Conclusion

Our study identified that the gut segments and DF co-contributed to the alterations of gut bacterial composition, resulting in their distinct metabolites. Several bacteria including *Bifidobacterium, Lactobacillus*, and *Streptococcus* may contribute to the high concentration of amino acids in the jejunum through their functions in protein digestion and absorption, and amino acid biosynthesis. The high concentration of free bile acids presented in the cecum, could be attributed to the *Lactobacilli, Bifidobacteria*, and *Streptococcus* enriched in jejunum, that function in deconjugating conjugated bile acids to form free bile acids that are resistant to reabsorption. Many fatty acids enriched in the cecum, which are positively linked with unidentified *Ruminococcaceae* and *Agathobacter*, serve in fatty acid biosynthesis, and biosynthesis of unsaturated fatty acids. DF significantly altered bacterial composition in both the cecum and jejunum. However, these variations only resulted in significant alterations in cecal metabolites. Carbohydrate metabolites including succinate, mannose 6-phosphate and D-galacturonic acid, were upregulated with increasing DF, which were closely related with Clostridia. The altered metabolites related to carbohydrate metabolism were mainly enriched in amino sugar and nucleotide sugar metabolism. DF increased the concentration of caprylic acid, undecanoic acid, arachidic acid, and dodecanoic acid which was positively linked to *Oscillibacter* through the KEGG functions pathway for fatty acid biosynthesis and biosynthesis of unsaturated fatty. Additionally, DF significantly decreased the concentration of bile acids, especially chenodeoxycholate, which is associated with the functions of primary bile acid biosynthesis, secondary bile acid biosynthesis, and bile secretion.

## Data Availability Statement

The datasets presented in this study can be found in online repositories. The names of the repository/repositories and accession number(s) can be found below: https://www.ncbi.nlm.nih.gov/, PRJNA685300.

## Ethics Statement

The animal study was reviewed and approved by Animal Ethics Committee of QingHai University (Approval Number: NQH2019102).

## Author Contributions

YZ and GW designed the study. GW, LZ, LW, and WS performed research and collected the samples. XT, YZ, LZ, GW, CF, and SR carried out the data analyses. XT and GW produced the initial draft of the manuscript. YZ, LZ, and CF contributed to the revision of the manuscript. All authors contributed to the article and approved the submitted version.

## Funding

This work was supported by the Key Research and Development Program of Qinghai Provincial Department of Science and Technology (No. 2021-QY-204); Second Tibetan Plateau Scientific Expedition and Research Program (No. 2019 QZKK0501); the National Natural Science Foundation of China (31670394); the project of western light for interdisciplinary team; and Science and Technology Department of Qinghai Province Major Project Sanjiangyuan National Park Animal Genome Program.

## Conflict of Interest

The authors declare that the research was conducted in the absence of any commercial or financial relationships that could be construed as a potential conflict of interest.

## Publisher's Note

All claims expressed in this article are solely those of the authors and do not necessarily represent those of their affiliated organizations, or those of the publisher, the editors and the reviewers. Any product that may be evaluated in this article, or claim that may be made by its manufacturer, is not guaranteed or endorsed by the publisher.

## Supplementary Material

Supplementary Table 1 Two-way ANOVA showing the effects of gastrointestinal tract and dietary fiber on the α-diversity of the core bacterial communities.
