# Supplementary material for: Gastrointestinal Tract and Dietary Fiber Driven Alterations of Gut Microbiota and Metabolites in Durco × Bamei Crossbred Pigs
Source: Front Nutr. 2022 Jan 28;8:806646. doi: 10.3389/fnut.2021.806646 (PMC8836464; doi:10.3389/fnut.2021.806646)
Supplement: Supplementary Table 1 — Two-way ANOVA showing the effects of gastrointestinal tract and dietary fiber on the α-diversity of the core bacterial communities. [file Table_1.docx]

Table S1. Two-way ANOVA showing the effects of gastrointestinal tract and dietary fiber on the α-diversity of core bacterial communities.

|  | Sums of squares | degree of freedom | Mean squares | *F* | *P* |
| --- | --- | --- | --- | --- | --- |
| Gastrointestinal tract |  |  |  |  |  |
| Shannon | 11.350 | 1 | 11.350 | 197.894 | **.000** |
| Simpson | .043 | 1 | .043 | 51.739 | **.000** |
|  |  |  |  |  |  |
| Fiber |  |  |  |  |  |
| Shannon | 2.261 | 3 | .754 | 13.143 | **.000** |
| Simpson | .011 | 3 | .004 | 4.654 | **.007** |
|  |  |  |  |  |  |
| Gastrointestinal tract & Fiber |  |  |  |  |  |
| Shannon | .626 | 3 | .209 | 3.636 | **.021** |
| Simpson | .005 | 3 | .002 | 1.987 | .131 |

*P* values < 0.05 in bold.
